# Effectiveness of a couple-based dyadic coping intervention on psychological distress and relationship functioning in HPV patients: a randomized controlled trial in China

**DOI:** 10.3389/fmed.2026.1867371

**Published:** 2026-09-11

**Authors:** Die Hou, Jiaxin He, Ruiqin Luo, Lihua Zhou

**Affiliations:** 1 Department of VIP Clinic Nursing, West China Second University Hospital, Sichuan University/West China School of Nursing, Sichuan University, Chengdu, Sichuan, China; 2 Ministry of Education, Key Laboratory of Birth Defects and Related Diseases of Women and Children (Sichuan University), Chengdu, Sichuan, China; 3 Department of Anesthesiology, Public Health Clinical Center of Chengdu, Chengdu, Sichuan, China; 4 Department of Neurology, Qionglai Medical Center Hospital, Qionglai, Sichuan, China

**Keywords:** couple-based intervention, dyadic coping, human papillomavirus, psychological distress, randomized controlled trial, relationship functioning

## Abstract

**Background:**

Human papillomavirus (HPV) infection may cause psychological distress and strain intimate relationships because concerns regarding transmission, stigma, and cancer risk can affect both patients and their partners. Evidence regarding HPV- specific couple-based psychosocial interventions remains limited.

**Objective:**

To evaluate the effects of a couple-based dyadic coping intervention on psychological distress and relationship functioning in patients with HPV infection.

**Methods:**

This randomized controlled trial enrolled 148 couples from a special-needs outpatient department, with the female partner in each couple diagnosed with HPV infection. Couples were randomly assigned in a 1:1 ratio to receive either an 8-week couple-based dyadic coping intervention plus routine care or routine care alone. The primary outcome was the change in the patient-reported Depression Anxiety Stress Scale-21 (DASS-21) total score from baseline to 8 weeks. Secondary outcomes included the HPV Impact Profile (HIP) total score and the patient- and spouse-reported Couples’ Illness Communication Scale (CICS), Dyadic Adjustment Scale (DAS), and Dyadic Coping Inventory (DCI) total scores. Follow-up compliance and HPV clearance were evaluated as exploratory outcomes. Between-group differences in changes were reported with 95% confidence intervals and Cohen’s *d*, and the robustness of the primary finding was further examined using multivariable ANCOVA and multiple imputation.

**Results:**

A total of 142 couples completed the 8-week assessment (intervention group, *n* = 72; control group, *n* = 70). The intervention group showed a greater reduction in the DASS-21 total score than the control group (difference in change, −4.98 points; 95% CI, −7.51 to −2.45; Cohen’s *d* = −0.65; *p <* 0.001). The adjusted ANCOVA and multiple-imputation sensitivity analysis yielded consistent findings. Secondary total-score outcomes also favored the intervention, with absolute standardized effect sizes ranging from 0.68 to 0.89 (all Holm-adjusted *p* < 0.001). Complete follow-up compliance was higher in the intervention group than in the control group (75.0% vs. 45.7%; *p* = 0.001). HPV clearance at 3 months was numerically higher in the intervention group (38.9% vs. 24.3%), but the between-group difference was not statistically significant (*p* = 0.062). No serious adverse events were reported.

**Conclusion:**

An 8-week couple-based dyadic coping intervention was associated with short-term improvements in psychological distress, HPV-specific impact, relationship functioning, dyadic coping, and follow-up compliance. The intervention shows promise as a supportive psychosocial approach but warrants confirmation in larger multicenter studies with longer follow-up before routine clinical implementation. The HPV clearance finding should be considered exploratory and hypothesis-generating.

## Introduction

Human papillomavirus (HPV) infection is one of the most common sexually transmitted infections worldwide (1). Although most HPV infections are transient and can be cleared by the immune system, persistent infection may lead to malignant transformation in the cervix, anus, larynx, and other sites (2). Because of the sexually transmitted nature of HPV infection and its potential cancer risks, HPV-infected patients often experience significant psychological distress, including anxiety, depression, social stigma, and concerns about sexual life (3). These psychological and interpersonal concerns may also affect intimate partners and place strain on couple communication, intimacy, and relationship functioning (4).

Stress within intimate relationships is inherently interdependent rather than solely individual. Stress experienced by one partner may spill over into couple interactions, impair communication, and reduce relationship satisfaction (5, 6). Dyadic coping theory, proposed by Bodenmann, emphasizes the importance of couples coping with stressors together as a functional unit (7). The theory suggests that effective dyadic coping includes emotional support, collaborative problem-solving, and joint meaning-making. When couples face health crises, their dyadic coping ability may influence disease adaptation and relationship quality (8). The Couples Coping Enhancement Training (CCET) operationalized this framework by integrating stress communication, supportive dyadic coping, and collaborative problem-solving (9). The present intervention shares these core components but was tailored to the HPV context by incorporating HPV-related health education, discussion of stigma and partner-transmission concerns, illness- and sexual-communication practice, joint coping exercises, homework assignments, and individualized telephone follow-up support.

Previous research on dyadic coping has shown encouraging findings across various health conditions, including cancer, multiple sclerosis, and cardiovascular disease (10–12). Couple-based interventions informed by this literature typically focus on improving communication skills, developing mutual support strategies, and enhancing collaborative problem-solving abilities. However, research on dyadic coping interventions specifically targeting couples dealing with HPV infection remains limited. Because of its sexually transmitted nature, HPV infection may create distinctive relational challenges, including partner blame, mistrust, sexual avoidance, and relationship conflict (13). The stigma associated with sexually transmitted infections may create psychological and relational stressors beyond those encountered in other chronic conditions, suggesting a need for interventions that address sexual concerns, partner transmission anxiety, and perceived social judgment. Moreover, a recent clinical review noted that, although management protocols for HPV-positive women are relatively well established, guidance for their male partners remains limited and controversial. The review highlighted the lack of standardized HPV testing and clear management pathways for male partners and proposed vaccination, counseling, and targeted clinical evaluation as possible components of partner care (14). These findings underscore that partner involvement in HPV management remains incompletely defined, particularly with regard to couple-level psychosocial adaptation. Psychological stress may also affect immune regulation, and observational evidence has linked stress with HPV-related immune responses and viral persistence (15–17). Thus, by reducing psychological distress and strengthening partner support, a dyadic intervention might indirectly influence stress-related immune regulation. Nevertheless, this represents only a plausible biological pathway and does not establish that a psychosocial intervention can improve HPV clearance. Therefore, HPV clearance was considered an exploratory, hypothesis-generating outcome in the present study.

This randomized controlled trial evaluated the effects of an 8-week couple-based dyadic coping intervention on psychological distress and relationship functioning among patients with HPV infection and their spouses. The primary hypothesis was that, compared with routine care, the intervention would produce a greater reduction in the patient-reported DASS-21 total score from baseline to 8 weeks. We further expected improvements in HPV-specific impact, illness communication, dyadic adjustment, and dyadic coping among patients and their spouses. Follow-up compliance and HPV clearance were evaluated as exploratory outcomes.

## Methods

### Study participants

This study was a two-arm, parallel, assessor-blinded randomized controlled trial with a 1:1 allocation ratio, comparing a couple-based dyadic coping intervention with routine care. The study population consisted of HPV-positive female patients and their spouses who attended the special-needs outpatient department of our hospital from March 2022 to February 2024. The special-needs outpatient department of our hospital, which is designed to cater to patients requiring enhanced psychosocial support and comprehensive health education alongside medical care, often due to the complex nature of their health condition (e.g., sexually transmitted infections).

Patients were eligible for inclusion if they met the following criteria: (1) aged 18–65 years; (2) high-risk HPV-DNA test positive with negative cervical liquid-based cytology; (3) time since HPV infection diagnosis ≤6 months, which targets the early post-diagnosis period when psychological distress and relationship disruption are typically most acute (18, 19); (4) stable heterosexual marriage ≥1 year, ensuring sufficient relationship foundation for meaningful participation in longitudinal dyadic research, consistent with sampling criteria in couple-based studies (20); (5) both patient and spouse voluntarily participated in the study and signed informed consent; and (6) normal cognitive function and communication ability.

Patients were excluded if they met any of the following criteria: (1) patient or spouse with other sexually transmitted diseases; (2) serious mental illness or cognitive impairment; (3) currently receiving other psychological or couple’s therapy; (4) marital relationship on the verge of breakdown or separation; (5) pregnant or lactating women; or (6) serious physical diseases affecting study participation.

Dropout criteria included: (1) missing more than 2 consecutive intervention sessions; (2) actively requesting to withdraw from the study; (3) divorce or separation during the study; or (4) loss to follow-up or inability to complete follow-up assessments.

This study was conducted in accordance with the Declaration of Helsinki and was approved by the Ethics Committee of West China Second University Hospital, Sichuan University (approval number: 2025416572289). All participants provided written informed consent before enrollment.

### Sample size calculation

The sample size calculation was based on the between-group difference in the change in patient-reported DASS-21 total score from baseline to 8 weeks, which was designated as the primary outcome. In the absence of directly comparable effect-size estimates for HPV-specific dyadic coping interventions, a moderate standardized effect size (Cohen’s *d* = 0.50) was selected as a pragmatic planning assumption. This assumed magnitude is consistent with the moderate effects reported in couple-based dyadic coping intervention studies conducted in other clinical populations (21, 22). Using G*Power version 3.1.9.7, with a two-sided significance level of *α* = 0.05, 80% power, and equal group allocation, 64 couples were required per group. To account for potential attrition, this number was increased by 15%, resulting in a target sample of 74 couples per group and 148 couples overall.

### Randomization and blinding

Block randomization was employed with a 1:1 allocation ratio to the intervention and control groups. Random sequences were generated by an independent statistician using SAS 9.4 software (SAS Institute Inc., Cary, NC, United States) with a block size of 4. To ensure allocation concealment, randomization assignments were placed in sequentially numbered, opaque, sealed envelopes. These envelopes were prepared by personnel not involved in participant recruitment or assessment and were stored in a locked cabinet accessible only to the research coordinator responsible for group assignment. After obtaining informed consent and completing baseline assessments, the research coordinator opened the next envelope in sequence to assign participants to their allocated group. Due to the behavioral nature of the intervention, participants and intervention facilitators could not be blinded to group allocation (assessor-blind design). However, outcome assessors who collected follow-up data at 8 weeks post-intervention were blinded to participants’ group assignments. To maintain assessor blinding, participants were instructed not to discuss their group allocation with outcome assessors, and assessment sessions were conducted separately from intervention sessions. The independent statistician who performed the data analysis was also blinded to group allocation until the completion of primary analyses. Group assignments were revealed only after all data collection and primary statistical analyses were completed.

### Intervention methods

Participants assigned to the control group received routine care, including HPV infection-related health education, regular follow-up examinations, medication guidance, and other standard care measures.

**Figure 1:**
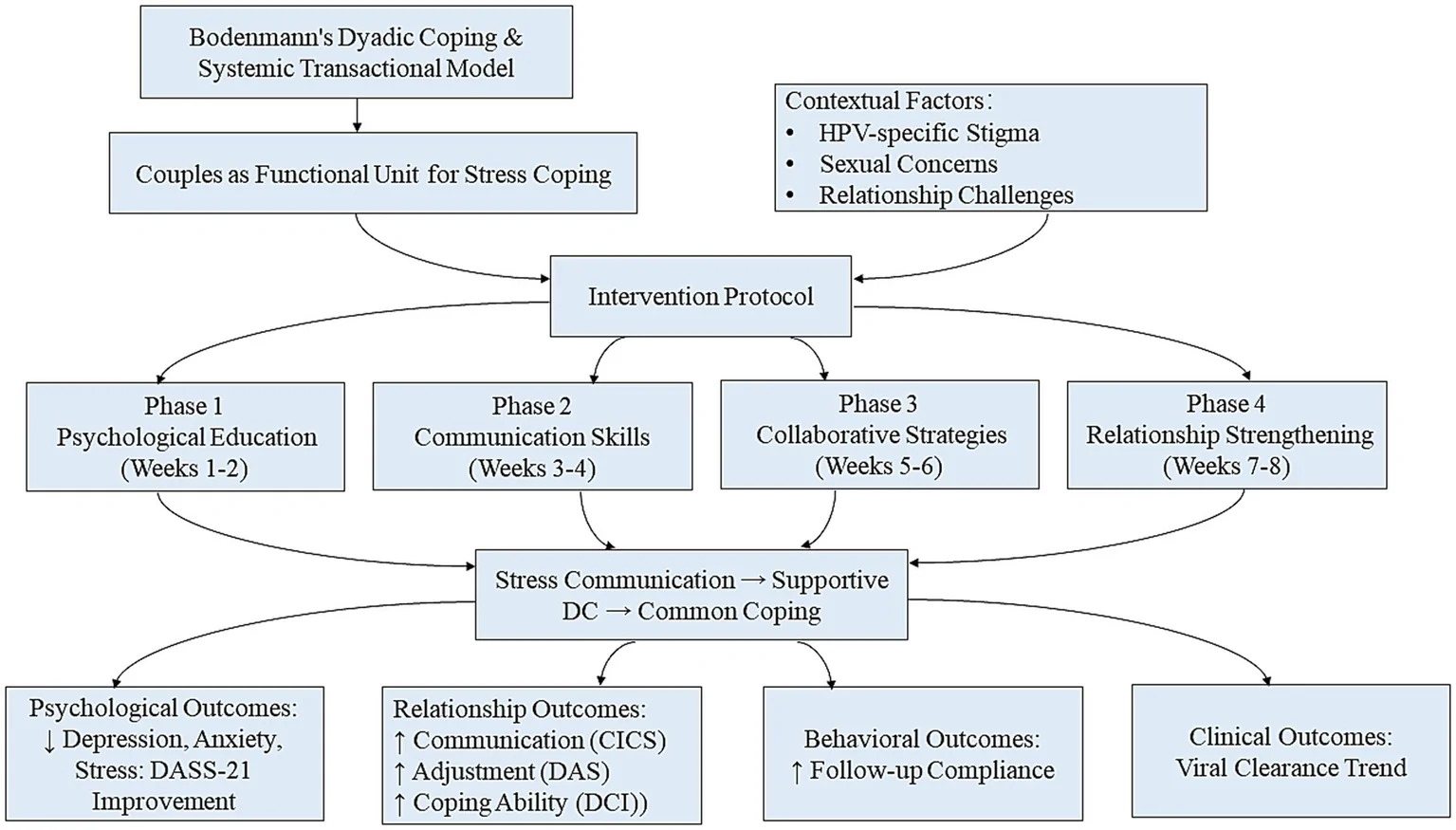
Conceptual framework of dyadic coping intervention for HPV-infected couples.

Participants assigned to the intervention group received an 8-week couple-based dyadic coping intervention in addition to routine care. The intervention protocol was designed based on Bodenmann’s dyadic coping theory and Systemic Transactional Model (7, 23), which emphasizes that effective coping with shared stressors requires sequential processes of stress communication, supportive dyadic coping, and common dyadic coping. The intervention is implemented by a team comprising two nursing specialists with psychotherapy qualifications and one gynecologist. The theoretical framework is presented in Figure 1. This theoretical framework was systematically operationalized through four sequential phases, each targeting specific mechanisms hypothesized to improve psychosocial outcomes: (1) Psychological Education Phase (Weeks 1–2): enhancing HPV knowledge and introducing dyadic coping concepts, with the theoretical aim of reducing negative dyadic coping (e.g., blame) by correcting misconceptions and establishing a foundation for effective stress communication, thereby hypothesized to decrease relationship conflict. (2) Communication Skills Training Phase (Weeks 3–4): improving couples’ communication quality through active listening, effective expression, and conflict resolution skills, which directly targets the core theoretical mechanism of stress communication to facilitate supportive dyadic coping responses, hypothesized to enhance couples’ communication and reduce psychological distress. (3) Collaborative Coping Strategy Development Phase (Weeks 5–6): developing personalized coping plans and emotion regulation techniques, operationalizing the theoretical construct of common dyadic coping through joint problem-solving. (4) Relationship Strengthening Training Phase (Weeks 7–8): rebuilding intimacy and establishing long-term relationship maintenance mechanisms, focusing on consolidating all positive dyadic coping forms to institutionalize new interaction patterns, hypothesized to sustain long-term relationship functioning. Detailed intervention content and activities are provided in Supplementary Table S1.

#### Implementation method

**Figure 2:**
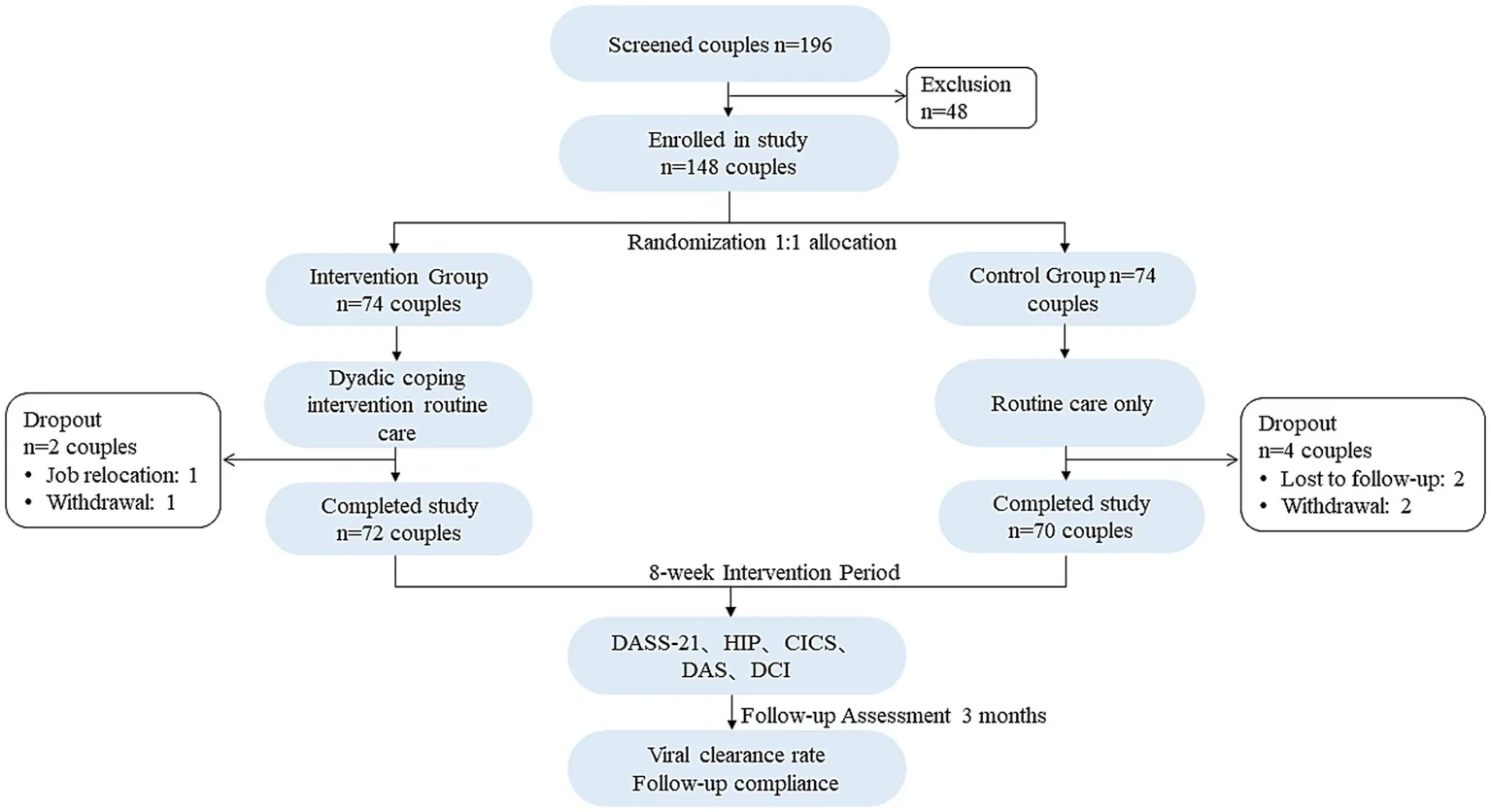
Study flowchart of couples-based dyadic coping intervention for HPV patients. A total of 196 couples were assessed for eligibility, of whom 48 were excluded. The remaining 148 couples were randomized in a 1:1 ratio to the intervention group or routine-care control group, with 74 couples initially allocated to each group. During the 8-week study period, two couples in the intervention group and four couples in the control group did not complete follow-up. Consequently, 72 and 70 couples, respectively, completed the 8-week assessment.

Weekly face-to-face group sessions (6–8 couples per group), 90 min each, including 30 min theoretical instruction, 45 min interactive practice, and 15 min summary sharing. Additionally, weekly 30-min individual telephone follow-up support was provided for personalized guidance on specific issues. Homework assignments including communication practice and coping strategy implementation were given after each session, with sharing and feedback in subsequent sessions (Figure 2).

### Intervention fidelity and quality control

To ensure intervention fidelity, all interventionists completed a 16-h standardized training program and delivered the intervention according to a detailed manual. After each group session and telephone follow-up, the interventionist completed a standardized checklist documenting participant attendance, session duration, delivery of the planned components, homework assignment, and any protocol deviations. Twenty percent of randomly selected sessions were audio-recorded and independently reviewed using the fidelity checklist, with a mean fidelity score of 94.3%. The completed checklists and any protocol deviations were reviewed during weekly supervision meetings, and corrective feedback was provided when necessary. Among the 72 couples in the intervention group, mean attendance was 7.6 ± 0.6 sessions out of eight, with 68 couples (94.4%) attending at least six sessions. The telephone follow-up completion rate was 98.6%.

### Outcome measures

Questionnaire-based outcomes were assessed at baseline and 8 weeks after the intervention. Viral clearance and follow-up compliance were assessed during the 3-month post-intervention follow-up.

### Primary outcome

#### Depression Anxiety Stress Scale-21

The primary outcome was the between-group difference in the change in patient-reported DASS-21 total score from baseline to 8 weeks. The scale contains 21 items divided into three dimensions: depression, anxiety, and stress, with 7 items each. Using 4-point Likert scoring, single dimension scores range from 0 to 21 points, total score 0–63 points, with higher scores indicating more severe psychological distress. The Chinese version has good reliability and validity, with Cronbach’s *α* coefficient of 0.959 (24, 25).

### Secondary outcomes

#### HPV impact profile

The HIP was completed by HPV-infected patients to assess the effects of HPV infection on their lives. The scale contains 29 items covering 7 dimensions: worry and concern, emotional impact, sexual impact, self-image, partner and transmission, interaction with healthcare providers, and life impact. Using 11-point Likert scoring, total scores are converted to standardized scores from 0 to 100 points, with higher scores indicating greater negative impact of HPV infection. The Chinese version of the HIP questionnaire was generated through a standard language validation procedure, the scale’s Cronbach’s *α* coefficient ranges from 0.64–0.90 (26, 27).

#### Couples’ Illness Communication Scale

The CICS was completed separately by patients and their spouses to assess communication about illness-related issues. The scale contains 4 items, each scored 1–5 points, total score 4–20 points, with higher scores indicating better couples’ illness communication levels. The Chinese version of the CICS showed excellent internal consistency, with Cronbach’s alpha values were 0.84 for patients and 0.80 for partners (28, 29).

#### Dyadic Adjustment Scale

The DAS was completed separately by patients and their spouses to assess overall relationship adjustment. The scale contains 32 items in 4 dimensions: Dyadic Consensus (13 items, 0–65 points), Dyadic Satisfaction (10 items, 0–50 points), Dyadic Cohesion (5 items, 0–24 points), and Affectional Expression (4 items, 0–12 points), total score 0–151 points, with higher scores indicating better relationship adjustment. The Chinese version of the DAS, which has been validated for use with Chinese couples, demonstrated excellent internal consistency, with coefficients of 0.93 for wives and 0.89 for husbands (30, 31).

#### Dyadic coping inventory

The DCI was completed separately by patients and their spouses at baseline and 8 weeks to assess dyadic coping. We used the validated Chinese version because the participants were Chinese-speaking couples. The Chinese version retains the conceptual domains and 5-point Likert response format (1 = very rarely to 5 = very often) of the original DCI. The original instrument contains 37 items, of which two global evaluation items are not included in the overall score. In the final factorial structure of the Chinese version, four stress-communication items were additionally omitted, resulting in 31 scored items. After reverse-scoring the Negative DC items, total scores range from 31 to 155, with higher scores indicating more adaptive dyadic coping. The 31 scored items cover seven dimensions: Stress Communication (4 items), Emotion-Focused Supportive DC (5 items), Problem-Focused Supportive DC (5 items), Delegated DC (4 items), Emotion-Focused Common DC (3 items), Problem-Focused Common DC (2 items), and Negative DC (8 items). The first six dimensions represent positive dyadic coping, whereas the Negative DC dimension represents negative dyadic coping. The Chinese version demonstrated good internal consistency, with Cronbach’s *α* coefficients of 0.85 for patients and 0.83 for their spouses (32, 33).

In the present study, Cronbach’s α was 0.90 for the DASS-21 total score and 0.89 for the HIP total score. For the dyadic measures, Cronbach’s α coefficients for patients and spouses were 0.83 and 0.81 for the CICS, 0.88 and 0.85 for the DAS, and 0.86 and 0.85 for the DCI, respectively.

### Exploratory follow-up outcomes

#### Viral clearance and follow-up compliance

Follow-up compliance was assessed based on the completion of scheduled visits within the prescribed follow-up intervals during the 3-month post-intervention period. Compliance was categorized as complete (100% timely completion), basic (≥80%), partial (60–79%), or non-compliance (<60%). Viral clearance was defined as two consecutive negative HPV-DNA test results obtained at least 4 weeks apart.

### Statistical analysis

Statistical analyses were performed using SPSS version 26.0 (IBM Corp., Armonk, NY, United Sates). The couple was the unit of randomization, and patient- and spouse-reported outcomes were analyzed separately. Continuous variables were expressed as mean ± standard deviation, and categorical variables as frequencies and percentages. Between-group comparisons of continuous variables were performed using independent-samples *t-*tests, whereas within-group changes from baseline to 8 weeks were assessed using paired-samples *t-*tests. Within-group comparisons were considered descriptive and were not used as the primary evidence of intervention effectiveness. Categorical variables were compared using Pearson’s chi-square test or Fisher’s exact test, as appropriate. Normality of continuous outcomes and change scores was assessed using the Shapiro–Wilk test, and homogeneity of variance for between-group comparisons was evaluated using Levene’s test.

For the primary outcome and the seven secondary total-score outcomes, individual change scores were calculated by subtracting baseline values from 8-week values and were compared between groups using independent-samples *t*-tests. These between-group comparisons of change scores were used to determine whether changes from baseline to 8 weeks differed between the intervention and control groups. Between-group differences in change were reported with 95% confidence intervals and Cohen’s *d*. The DASS-21 total score was designated as the primary outcome and was evaluated using a two-sided *α* of 0.05 without adjustment for multiple comparisons. Holm correction was applied across the seven secondary total-score comparisons. Subscale analyses were considered exploratory; no multiplicity adjustment was applied to these analyses, and the corresponding unadjusted *p-*values were reported descriptively and interpreted with caution.

The primary complete-case analyses included 142 couples with both baseline and 8-week assessments (72 in the intervention group and 70 in the control group). Multiple imputation was additionally performed as a sensitivity analysis to assess the potential influence of missing 8-week data. A multivariable ANCOVA was conducted for the 8-week DASS-21 total score, with treatment group as the main independent variable and baseline DASS-21 score, age, education level, relationship duration, and HPV infection type as covariates.

Exploratory partial Pearson correlation analyses were conducted among baseline-to-8-week changes in the patient-reported DASS-21, HIP, CICS, DAS, and DCI total scores, controlling for treatment group. Holm correction was applied to the 10 pairwise correlation tests as a separate family of comparisons. Internal consistency of the psychometric instruments in the present sample was evaluated using Cronbach’s *α*. All tests were two-sided, and *p* < 0.05 was considered statistically significant; Holm-adjusted *p-*values were used where multiplicity correction was specified. Secondary total-score, subscale, and correlation analyses were interpreted as exploratory.

## Results

### Participant characteristics

A total of 196 couples were subjected to a screening process, out of which 48 couples were excluded on the basis of nonconformity with the established inclusion criteria. Consequently, 148 couples were ultimately enrolled. During the 8-week intervention period, 2 couples dropped out from the intervention group (1 due to job relocation, 1 active withdrawal) and 4 couples from the control group (2 lost to follow-up, 2 active withdrawals). Finally, 142 couples completed the study (72 couples in intervention group, 70 couples in control group), with a dropout rate of 4.1%. As shown in Table 1, baseline characteristic comparisons between groups showed no statistical differences in any indicators (*p* > 0.05), demonstrating good comparability.

**Table 1:** Comparison of baseline characteristics between groups.

Characteristics	Intervention group (*n* = 72)	Control group (*n* = 70)	Statistics	*p-*value
Patient characteristics				
Age (years, mean ± SD)	32.84 ± 6.73	33.41 ± 7.12	0.490	0.625
Education level [n(%)]			0.098	0.952
Junior high or below	10 (13.9)	9 (12.9)		
High school/vocational	21 (29.2)	22 (31.4)		
College or above	41 (56.9)	39 (55.7)		
Employment status [n(%)]			0.834	0.659
Enterprise/institution	38 (52.8)	32 (45.7)		
Self-employed	21 (29.2)	25 (35.7)		
Other	13 (18.1)	13 (18.6)		
HPV infection type [n(%)]			0.006	0.938
Single infection	51 (70.8)	50 (71.4)		
Multiple infection	21 (29.2)	20 (28.6)		
HPV subtype distribution [n(%)]			1.824	0.935
HPV16	18 (25.0)	16 (22.9)		
HPV18	9 (12.5)	10 (14.3)		
HPV52	15 (20.8)	14 (20.0)		
HPV58	12 (16.7)	13 (18.6)		
Other high-risk types*	18 (25.0)	17 (24.3)		
Partner characteristics				
Age (years, mean ± SD)	34.67 ± 7.28	35.23 ± 7.65	0.447	0.656
Education level [n(%)]			0.276	0.871
Junior high or below	10 (13.9)	9 (12.9)		
High school/vocational	24 (33.3)	21 (30.0)		
College or above	38 (52.8)	40 (57.1)		
Employment status [n(%)]			0.405	0.817
Enterprise/institution	40 (55.6)	36 (51.4)		
Self-employed	24 (33.3)	24 (34.3)		
Other	8 (11.1)	10 (14.3)		
Relationship characteristics				
Relationship duration (years, mean ± SD)	6.74 ± 2.26	6.35 ± 2.43	0.991	0.324
Having children [n(%)]	45 (62.5)	41 (58.6)	0.229	0.632
Monthly household income [yuan, M(Q1, Q3)]	6,647 (4,785, 9,136)	6,782 (4,893, 9,274)	0.249	0.803

*Other high-risk types include HPV31, 33, 35, 39, 45, 51, 56, 59, 66, 68, etc.

### Changes in patient psychological distress levels

#### DASS-21 scale assessment results

**Figure 3:**
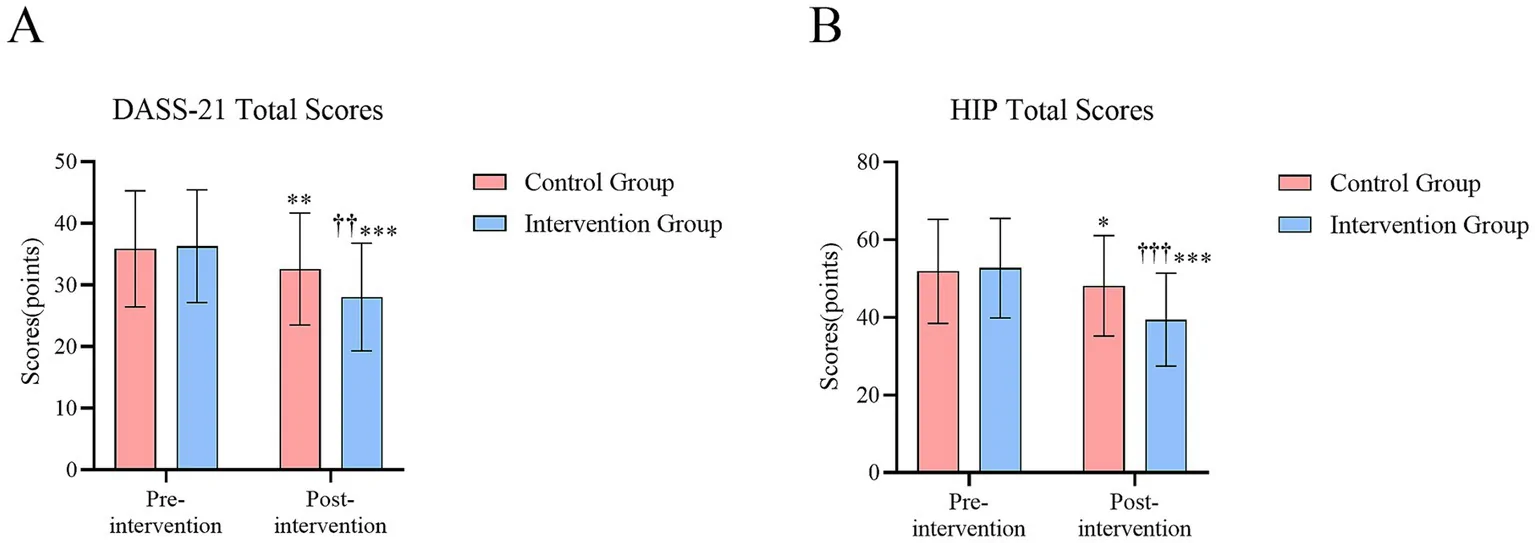
Changes in psychological distress and HPV-specific impact total scores. **(A)** DASS-21 total scores. **(B)** HIP total scores. Values are presented as mean ± standard deviation. ††*p* < 00.01, †††*p* < 0.001 for between-group comparisons; *p < 0.05, **p < 0.01, ***p < 0.001 compared with same group pre-intervention. DASS-21, Depression Anxiety Stress Scale-21; HIP, HPV Impact Profile.

No statistical differences were found in DASS-21 scores between groups pre-intervention (*p* > 0.05). Post-intervention, the intervention group exhibited significantly lower total DASS-21 scores compared to the control group post-intervention (*p* < 0.001), as illustrated in Figure 3A. Subscale analyses are presented in Table 2. Between-group comparisons revealed that the intervention group achieved significantly lower scores across all three dimensions, including depression, anxiety, and stress, compared to the control group (*p* < 0.05).

**Table 2:** Comparison of DASS-21 scale scores between groups (mean ± SD, points).

Indicator	Intervention group (*n* = 72)	Control group (*n* = 70)	*t-*value	*p-*value
Depression dimension				
Pre-intervention	9.84 ± 3.42	9.51 ± 3.68	0.554	0.581
Post-intervention	7.36 ± 3.18***	9.12 ± 3.45	3.162	0.002
Anxiety dimension				
Pre-intervention	13.72 ± 3.85	13.48 ± 3.92	0.368	0.713
Post-intervention	10.44 ± 3.64***	11.89 ± 3.67***	2.364	0.020
Stress dimension				
Pre-intervention	12.72 ± 3.45	12.85 ± 3.58	0.220	0.826
Post-intervention	10.24 ± 3.21***	11.57 ± 3.42**	2.390	0.018

Data are presented as mean ± standard deviation. **p < 00.01, ***p < 0.001 compared with pre-intervention within the same group. DASS-21, Depression Anxiety Stress Scale-21.

### HPV-specific impact assessment results

No statistical differences were found in HIP scores between groups pre-intervention (*p* > 0.05). Post-intervention, the intervention group exhibited significantly lower HIP total scores compared to the control group (*p* < 0.05), as illustrated in Figure 3B. Detailed dimensional analyses are presented in Table 3. Between-group comparisons revealed that the intervention group achieved significantly lower scores across six dimensions, including worry and concern, emotional impact, sexual impact, self-image, partner and transmission, and life impact, compared to the control group (*p* < 0.01). No significant between-group difference was observed in the “interaction with healthcare providers” dimension (*p* > 0.05).

**Table 3:** Comparison of HIP Scale Scores between groups (mean ± SD, points).

Indicator	Intervention group (*n* = 72)	Control group (*n* **=** 70)	*t-*value	*p-*value
Worry and concern				
Pre-intervention	57.83 ± 14.27	56.94 ± 15.05	0.362	0.718
Post-intervention	40.42 ± 13.68***	51.17 ± 14.38**	4.565	<0.001
Emotional impact				
Pre-intervention	54.29 ± 13.74	53.67 ± 14.19	0.265	0.792
Post-intervention	41.18 ± 12.95***	52.42 ± 13.56	5.052	<0.001
Sexual impact				
Pre-intervention	49.65 ± 15.83	48.92 ± 16.27	0.271	0.787
Post-intervention	38.74 ± 14.47***	47.38 ± 15.69	3.413	0.001
Self-image				
Pre-intervention	51.36 ± 14.52	50.78 ± 15.13	0.233	0.816
Post-intervention	38.29 ± 13.86***	45.64 ± 14.28**	3.112	0.002
Partner and transmission				
Pre-intervention	55.72 ± 16.94	54.86 ± 17.45	0.298	0.766
Post-intervention	42.48 ± 15.72***	51.19 ± 16.83	3.188	0.002
Interaction with healthcare providers				
Pre-intervention	46.83 ± 18.26	45.97 ± 18.74	0.277	0.782
Post-intervention	36.65 ± 16.68***	42.23 ± 17.95	1.920	0.057
Life impact				
Pre-intervention	48.92 ± 14.37	48.15 ± 15.02	0.312	0.755
Post-intervention	38.46 ± 13.93***	47.07 ± 14.26	3.640	<0.001

Data are presented as mean ± standard deviation. **p < 00.01, ***p < 0.001 compared with pre-intervention within the same group. HIP, Human Papillomavirus Impact Profile.

### Changes in relationship functioning

#### Couples’ illness communication assessment results

**Figure 4:**
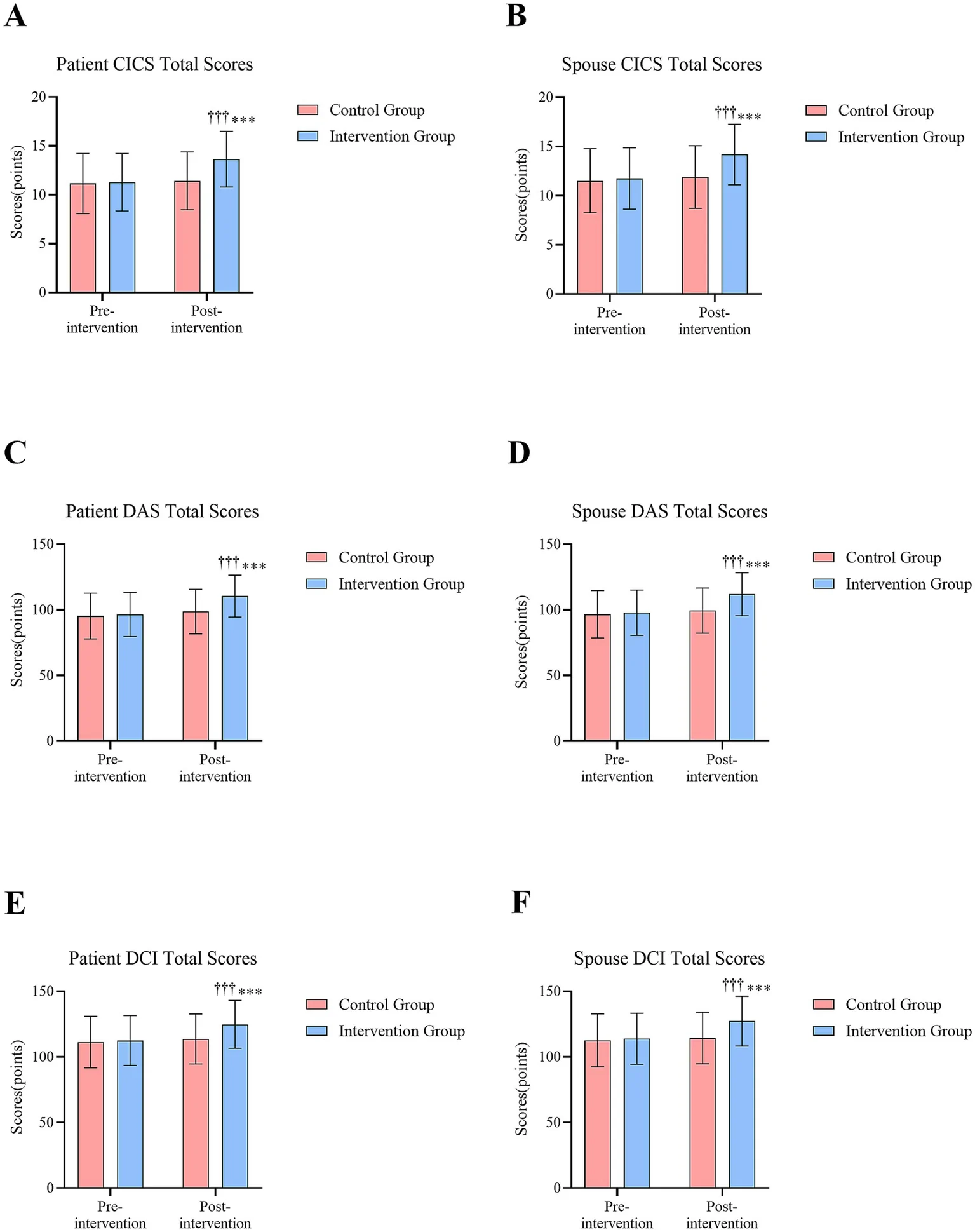
Changes in relationship functioning and dyadic coping total scores. **(A)** Patient CICS total scores. **(B)** Spouse CICS total scores. **(C)** Patient DAS total scores. **(D)** Spouse DAS total scores. **(E)** Patient DCI total scores. **(F)** Spouse DCI total scores. Data are presented as mean ± standard deviation for pre-intervention and post-intervention assessments. ***p < 0.001 compared with pre-intervention within the same group; †††*p* < 0.001 for between-group comparisons. CICS, Couples’ Illness Communication Scale; DAS, Dyadic Adjustment Scale; DCI, Dyadic Coping Inventory.

No statistical differences were found in CICS scores between groups pre-intervention (*p* > 0.05). Post-intervention, both patients and spouses in the intervention group had significantly higher CICS scores than the control group (*p* < 0.001). Results are presented in Figures 4A,B.

### Relationship adjustment assessment results

No statistical differences were found in DAS scores between groups pre-intervention (*p* > 0.05). Post-intervention, both patients and spouses in the intervention group had significantly higher DAS total scores compared to the control group (*p* < 0.001), as illustrated in Figures 4C,D respectively. Detailed dimensional analyses are presented in Table 4. Between-group comparisons revealed that both patients and spouses in the intervention group achieved significantly higher scores across all four dimensions, including dyadic consensus, dyadic satisfaction, dyadic cohesion, and affectional expression, compared to the control group (*p* < 0.05).

**Table 4:** Comparison of DAS scale scores between groups (mean ± SD, points).

Indicator	Intervention group (*n* = 72 couples)	Control group (*n* = 70 couples)	*t*-value	*p-*value
Patient dyadic consensus				
Pre-intervention	42.68 ± 8.95	42.14 ± 9.27	0.353	0.725
Post-intervention	47.32 ± 8.68***	43.06 ± 8.84	2.897	0.004
Spouse dyadic consensus				
Pre-intervention	43.25 ± 9.14	42.71 ± 9.48	0.346	0.729
Post-intervention	48.29 ± 8.95***	43.68 ± 9.12	3.04	0.003
Patient dyadic satisfaction				
Pre-intervention	32.74 ± 6.82	32.29 ± 7.15	0.384	0.702
Post-intervention	37.18 ± 6.94***	33.17 ± 6.83	3.469	0.001
Spouse dyadic satisfaction				
Pre-intervention	33.18 ± 7.03	32.84 ± 7.29	0.283	0.778
Post-intervention	37.53 ± 6.97***	33.65 ± 6.95	3.321	0.001
Patient dyadic cohesion				
Pre-intervention	13.86 ± 4.26	13.52 ± 4.48	0.464	0.644
Post-intervention	16.91 ± 4.15***	14.37 ± 4.29	3.586	<0.001
Spouse dyadic cohesion				
Pre-intervention	14.12 ± 4.37	13.78 ± 4.52	0.456	0.649
Post-intervention	17.26 ± 4.22***	14.55 ± 4.36	3.764	<0.001
Patient affectional expression				
Pre-intervention	7.19 ± 2.85	7.34 ± 2.94	0.309	0.758
Post-intervention	9.12 ± 2.76***	8.16 ± 2.87	2.032	0.044
Spouse affectional expression				
Pre-intervention	7.28 ± 2.91	7.34 ± 3.02	0.121	0.904
Post-intervention	8.86 ± 2.68***	7.54 ± 2.75	2.897	0.004

Data are presented as mean ± standard deviation. ***p < 0.001 compared with pre-intervention within the same group. DAS, Dyadic Adjustment Scale.

### Dyadic coping ability assessment results

No statistical differences were found in DCI scores between groups pre-intervention (*p* > 0.05). Post-intervention, both patients and spouses in the intervention group exhibited significantly higher DCI total scores compared to the control group (*p* < 0.001), as illustrated in Figures 4E,F respectively. Detailed dimensional analyses are presented in Table 5. Post-intervention, both patients and spouses in the intervention group had significantly higher DCI total scores and positive dyadic coping dimension scores compared to the control group (*p* < 0.001), with significant improvements in negative dyadic coping dimension scores as well.

**Table 5:** Comparison of DCI scale scores between groups (mean ± SD, points).

Indicator	Intervention group (*n* = 72 couples)	Control group (*n* = 70 couples)	*t*-value	*p-*value
Patient positive dyadic coping				
Pre-intervention	89.23 ± 15.68	88.47 ± 16.24	0.284	0.777
Post-intervention	99.86 ± 15.32***	90.15 ± 15.87	3.710	<0.001
Spouse positive dyadic coping				
Pre-intervention	90.67 ± 16.15	89.84 ± 16.75	0.301	0.764
Post-intervention	102.42 ± 15.68***	91.78 ± 16.29	3.966	<0.001
Patient negative dyadic coping				
Pre-intervention	23.24 ± 4.87	22.82 ± 5.14	0.500	0.618
Post-intervention	26.58 ± 4.73***	22.96 ± 4.92	4.470	<0.001
Spouse negative dyadic coping				
Pre-intervention	23.22 ± 4.93	22.83 ± 5.07	0.465	0.643
Post-intervention	26.84 ± 4.68***	23.14 ± 4.85	4.626	<0.001

Data are presented as mean ± standard deviation. ***p < 0.001 compared with pre-intervention within the same group. DCI, Dyadic Coping Inventory.

### HPV viral clearance rate and follow-up compliance assessment results

Follow-up compliance was significantly better in the intervention group than in the control group (*p* < 0.05). As shown in Table 6, complete compliance was reported for 75.0% of patients in the intervention group compared with 45.7% in the control group, whereas non-compliance was reported for 2.8 and 18.6%, respectively. At 3 months post-intervention, the HPV clearance rate was numerically higher in the intervention group than in the control group; however, the difference was not statistically significant (*p* = 0.062). Therefore, the HPV clearance result was considered exploratory and hypothesis-generating and should not be interpreted as evidence of intervention efficacy for viral clearance.

**Table 6:** Comparison of viral clearance rate and follow-up compliance between groups.

Indicator	Intervention group (*n* = 72)	Control group (*n* = 70)	χ^2^ value	*p-*value
Viral clearance rate [n(%)]				
Post-intervention (immediate)	18 (25.0)	12 (17.1)	1.315	0.251
3 months post-intervention	28 (38.9)	17 (24.3)	3.496	0.062
Follow-up compliance [n (%)]				
Complete compliance	54 (75.0)	32 (45.7)		
Basic compliance	12 (16.7)	18 (25.7)	15.690	0.001
Partial compliance	4 (5.6)	7 (10.0)		
Non-compliance	2 (2.8)	13 (18.6)		

Data are presented as n (%). Between-group comparisons were performed using the Chi-square test.

### Between-group comparisons of changes and sensitivity analyses

Individual change-score analyses showed that the reduction in DASS-21 total score was greater in the intervention group than in the control group (mean change, −8.24 ± 7.49 versus −3.26 ± 7.75; between-group difference in change, −4.98 points; 95% CI, −7.51 to −2.45; t (140) = −3.89; *p* < 0.001; Cohen’s *d* = −0.65). A greater reduction in HIP total score was also observed in the intervention group (difference in change, −9.55 points; 95% CI, −13.11 to −5.99; *p* < 0.001; Cohen’s *d* = −0.89).

Compared with routine care, the intervention group showed greater improvements in patient and spouse CICS total scores, with differences in change of 2.09 points (95% CI, 1.27–2.91) and 2.06 points (95% CI, 1.18–2.94), respectively. Corresponding differences were 10.59 points (95% CI, 6.26–14.92) and 11.36 points (95% CI, 6.92–15.80) for patient and spouse DAS total scores. Patient and spouse DCI total scores showed differences in change of 12.15 points (95% CI, 6.23–18.07) and 13.12 points (95% CI, 7.45–18.79), respectively. All seven secondary total-score comparisons remained statistically significant after Holm correction (all adjusted *p* < 0.001) (Table 7).

**Table 7:** Between-group comparisons of changes in primary and secondary total-score outcomes.

Outcome	Intervention Δ (*n* = 72)	Control Δ (*n* = 70)	Difference in change (95% CI)	*t*	*p*	Cohen’s *d*
DASS-21 total	−8.24 ± 7.49	−3.26 ± 7.75	−4.98 (−7.51, −2.45)	−3.89	<0.001	−0.65
HIP total	−13.28 ± 10.42	−3.73 ± 11.04	−9.55 (−13.11, −5.99)	−5.30	<0.001	−0.89
Patient CICS total	2.36 ± 2.42	0.27 ± 2.52	2.09 (1.27, 2.91)	5.04	<0.001	0.85
Spouse CICS total	2.44 ± 2.59	0.38 ± 2.70	2.06 (1.18, 2.94)	4.64	<0.001	0.78
Patient DAS total	14.06 ± 12.72	3.47 ± 13.33	10.59 (6.26, 14.92)	4.84	<0.001	0.81
Spouse DAS total	14.11 ± 13.05	2.75 ± 13.70	11.36 (6.92, 15.80)	5.06	<0.001	0.85
Patient DCI total	13.97 ± 17.50	1.82 ± 18.20	12.15 (6.23, 18.07)	4.06	<0.001	0.68
Spouse DCI total	15.37 ± 16.80	2.25 ± 17.40	13.12 (7.45, 18.79)	4.57	<0.001	0.77

Change scores (Δ) are presented as mean ± SD and were calculated as the 8-week score minus the baseline score. Differences in change were calculated as the intervention-group change minus the control-group change. The *p*-value for the primary DASS-21 outcome was unadjusted, whereas the *p*-values for the seven secondary total-score outcomes were adjusted using the Holm method as a single family of comparisons. Cohen’s *d* was calculated using the pooled SD of the change scores. Negative changes in DASS-21 and HIP scores and positive changes in CICS, DAS, and DCI scores indicate improvement. DASS-21, Depression Anxiety Stress Scale-21; HIP, HPV Impact Profile; CICS, Couples’ Illness Communication Scale; DAS, Dyadic Adjustment Scale; DCI, Dyadic Coping Inventory; CI, confidence interval; SD, standard deviation.

The Shapiro–Wilk and Levene’s tests indicated no substantial violations of the normality or homogeneity-of-variance assumptions. In the multivariable ANCOVA, the intervention remained associated with a lower 8-week DASS-21 total score after adjustment for baseline DASS-21 score, age, education level, relationship duration, and HPV infection type [adjusted mean difference, −4.89 points; 95% CI, −7.17 to −2.61; *F* (1, 134) = 17.96; *p* < 0.001; partial η^2^ = 0.118]. The multiple-imputation sensitivity analysis produced a consistent adjusted estimate (−4.77 points; 95% CI, −7.05 to −2.49; *p* < 0.001).

### Adverse events

No serious adverse events related to the study occurred in either group during the entire study period. Three couples in the intervention group reported mild emotional fluctuations during initial participation, which were resolved with psychological support and they continued to complete the study.

### Correlations among changes in psychological and dyadic outcomes

Exploratory partial Pearson correlation analyses were conducted among changes in the patient-reported outcome measures, controlling for treatment group. All reported *p-*values were adjusted for the 10 pairwise correlations using the Holm method. Reductions in DASS-21 scores were associated with reductions in HIP scores (partial *r* = 0.42, *p* < 0.001) and increases in CICS (partial *r* = −0.27, *p* = 0.005), DAS (partial *r* = −0.34, *p* < 0.001), and DCI scores (partial *r* = −0.38, *p* < 0.001). Reductions in HIP scores were also associated with increases in DAS (partial *r* = −0.21, *p* = 0.026) and DCI scores (partial *r* = −0.25, *p* = 0.009), whereas the association with CICS scores was not statistically significant (partial *r* = −0.14, *p* = 0.100). Improvements in CICS, DAS, and DCI scores were positively correlated with one another (partial *r* = 0.29–0.46, all *p* ≤ 0.003). These exploratory findings indicate that improvements in psychological distress and dyadic functioning were related, although causal or mediating relationships cannot be inferred.

## Discussion

This randomized controlled trial found that, compared with routine care, the 8-week couple-based dyadic coping intervention was associated with reduced patient-reported psychological distress and improvements in HPV-specific impact, illness communication, dyadic adjustment, and dyadic coping. The primary finding remained consistent in the multivariable and missing-data sensitivity analyses. Improvements were also observed across several patient- and spouse-reported secondary outcomes, although the secondary and subscale findings should be interpreted as exploratory. Follow-up compliance was higher in the intervention group, while HPV clearance was numerically higher but did not reach statistical significance.

The overall pattern of findings is consistent with relational stress theory and Bodenmann’s systemic-transactional model, which conceptualize stress and coping as interdependent processes within couples (5, 6). Within this framework, HPV-related stress may vary in its source, duration, and intensity. An HPV diagnosis and the associated medical demands may initially constitute an external, acute, and potentially major stressor for the couple. Persistent infection and repeated surveillance may subsequently develop into a chronic burden, whereas recurring concerns about disclosure, transmission, sexual activity, and follow-up may operate as cumulative minor stressors. When these pressures spill over into blame, mistrust, sexual avoidance, or impaired communication, an external health-related stressor may become an internal relational stressor. Accordingly, the intervention’s emphasis on stress communication, supportive dyadic coping, and common dyadic coping was intended to help couples address both the medical demands of HPV infection and their relational consequences. Systematic reviews have similarly found that positive dyadic coping is generally associated with more favorable individual and relational outcomes, whereas negative dyadic coping shows the opposite pattern (34, 35). Improvements in both patient- and spouse-reported outcomes in the present study are therefore compatible with a couple-level interpretation.

The exploratory partial correlation analyses provided additional, although non-causal, support for this interpretation. After adjustment for treatment group, reductions in DASS-21 scores were associated with reductions in HIP scores (partial *r* = 0.42) and improvements in CICS, DAS, and DCI scores (partial *r* ranging from − 0.27 to − 0.38). These findings indicate that improvements in psychological distress and relationship functioning occurred together. However, correlations between changes measured over the same two assessment points cannot establish directionality or demonstrate that improvements in communication or dyadic coping mediated the intervention effect. Formal longitudinal mediation analysis would be required to evaluate these mechanisms.

The present intervention shared several core components with the Couples Coping Enhancement Training, including stress communication, supportive coping, collaborative problem-solving, and joint coping practice (9). Its contribution lies primarily in adapting these established components to the HPV context rather than proposing a new model of dyadic coping. The direction of the present findings is consistent with a randomized study of a couple-based dyadic coping intervention among colorectal cancer patient–spouse dyads, although the study population and intervention protocol differed (10). Research involving couples affected by multiple sclerosis and cardiovascular disease has also linked dyadic coping with care experiences and health-related adjustment (11, 12).

HPV infection may present distinctive relational challenges that are less prominent in many other health conditions. Women with HPV have reported concerns about sexual relationships, disclosure, transmission, and possible negative reactions from their partners (3, 4). Stigmatizing beliefs may further contribute to shame, fear of judgment, and reluctance to discuss the infection openly (36, 37). HPV diagnosis has also been associated with anxiety and sexual dysfunction, while stigma remains common among women with high-risk HPV infection (38, 39). The present intervention directly addressed these concerns through HPV-related education, therapist-facilitated communication, joint coping exercises, and follow-up support. Psychoeducation may have reduced misconceptions and blame, while communication practice may have helped couples discuss transmission and sexual concerns more constructively. However, because the intervention components were not evaluated separately, these possible mechanisms remain interpretive rather than confirmed.

Follow-up behavior was another potentially important finding. Complete compliance was reported by 75.0% of patients in the intervention group compared with 45.7% in the control group. Improved disease knowledge, partner support, and structured follow-up planning may have contributed to this difference. However, these pathways were not directly evaluated, and the greater amount of professional contact received by the intervention group may also have produced attention or reminder effects.

At 3 months, HPV clearance was observed in 38.9% of the intervention group and 24.3% of the control group, but the between-group difference was not statistically significant (*p* = 0.062). This finding should therefore be considered exploratory and hypothesis-generating and should not be interpreted as evidence that the intervention improved HPV clearance. Psychological stress has been associated with changes in human immune function, providing a general biological rationale for further investigation (15). HPV-specific literature has also reported possible associations between psychological stress and HPV manifestations or viral persistence (16, 17). However, the present study did not measure stress-related hormones, immune biomarkers, or HPV viral load and was not powered to detect a difference in HPV clearance. No biological effect or psychoneuroimmunological mechanism can therefore be inferred from these data.

Several limitations should be considered. Recruitment from a single special-needs outpatient department and the restriction of the sample to heterosexual couples with an HPV-positive female partner may limit the generalizability of the findings to patients in general gynecology clinics, male HPV-positive patients, same-sex couples, and populations with different healthcare access or sociocultural backgrounds. Participants and interventionists could not be blinded, and the routine-care control condition did not match the intervention for professional contact time; therefore, expectation, attention, and performance effects may have contributed to the observed differences. The psychological and relational outcomes were self-reported, and responses concerning sexual health and intimate relationships may have been affected by recall and social desirability biases, particularly in face-to-face clinical assessments. In addition, psychosocial outcomes were assessed only at baseline and 8 weeks, while the 3-month follow-up was insufficient to determine the durability of the intervention effects or their relationship with HPV clearance. Although multiplicity adjustment and sensitivity analyses were applied, the secondary, subscale, and correlation analyses should still be interpreted as exploratory.

Within these limitations, the findings provide preliminary evidence that an HPV-specific couple-based dyadic coping intervention may reduce short-term psychological distress, improve relationship functioning, and support follow-up behavior. Larger, prospectively registered multicenter trials with active attention-control conditions and longer follow-up are needed to confirm these findings and evaluate their durability. Future studies should include more diverse couples and prospectively specify a primary outcome and analysis plan. Studies examining potential mechanisms should incorporate longitudinal mediation analyses and relevant behavioral or immunological measurements. Implementation research addressing feasibility, fidelity, resource requirements, and scalability would also be required before routine clinical adoption could be recommended.

## Conclusion

This study provides preliminary evidence that an 8-week couple-based dyadic coping intervention was associated with reduced psychological distress and improvements in HPV-specific impact, illness communication, dyadic adjustment, dyadic coping, and follow-up compliance among patients with HPV infection and their spouses. The between-group difference in HPV clearance was not statistically significant and should be considered exploratory and hypothesis-generating. Given the single-center design and short follow-up period, the intervention shows promise as a supportive psychosocial approach but warrants further validation in larger multicenter studies with longer follow-up before routine clinical implementation.

## Data Availability

The original contributions presented in the study are included in the article/Supplementary material, further inquiries can be directed to the corresponding author.
