# Supplementary material for: Effectiveness of a couple-based dyadic coping intervention on psychological distress and relationship functioning in HPV patients: a randomized controlled trial in China
Source: Front Med (Lausanne). 2026 Sep 11;13:1867371. doi: 10.3389/fmed.2026.1867371 (PMC13612200; doi:10.3389/fmed.2026.1867371)
Supplement: Supplementary file 1 [file Table_1.DOCX]

**Supplementary Table S1. Detailed 8-Week Couple-Based Dyadic Coping Intervention Protocol**

| Week | Phase | Session Objectives | Main Content | Key Activities | Homework Assignments |
| --- | --- | --- | --- | --- | --- |
| 1-2 | Psychological Education Phase | • Enhance scientific understanding of HPV • Reduce anxiety and misconceptions • Introduce dyadic coping framework | • HPV epidemiology, transmission routes, and natural history • Differences between high-risk and low-risk HPV types • HPV-related disease risks and cancer prevention • Importance of vaccination and regular screening • Clarifying common misconceptions (e.g., "HPV infection inevitably leads to cancer," "infection means partner infidelity") • Introduction to dyadic coping theory and couple collaboration | • Multimedia presentations • Case analysis and discussion • Interactive Q&A sessions • Small group discussions • Myth-busting exercises | • Review HPV educational materials together • Discuss and share concerns about HPV diagnosis • Identify personal misconceptions |
| 3-4 | Communication Skills Training Phase | • Improve couple communication quality • Enhance mutual understanding and support • Master conflict resolution skills | • Active listening skills: focused listening, feedback confirmation, emotional empathy • Effective expression training: using "I" statements, clarifying needs, non-accusatory communication • Conflict resolution skills: identifying triggers, pause techniques, seeking win-win solutions • Emotional support skills: emotional validation, comfort strategies, encouragement techniques • Sexual health communication: openly discussing sexual concerns, developing safe sex strategies | • Role-playing exercises • Scenario simulation (conflict situations) • Dyadic communication practice • Video demonstrations • Peer feedback sessions | • Practice 15-minute daily couple communication • Use "I" statements in discussions • Complete communication log • Practice one conflict resolution technique |
| 5-6 | Collaborative Coping Strategy Development Phase | • Develop personalized coping plans • Enhance coping confidence • Master stress management techniques | • Stressor identification: systematically identifying physical, psychological, and social stressors from HPV infection • Coping resource assessment: identifying personal strengths, family support, social resources • Problem-solving strategies: analyzing specific problems, developing action plans, task division • Emotion regulation techniques: deep breathing training, progressive muscle relaxation, mindfulness meditation • Social support utilization: seeking professional help, peer support, family understanding • Crisis response planning: developing plans for abnormal test results, disease progression | • Stressor mapping exercise • Resource inventory worksheet • Problem-solving workshop • Relaxation technique training • Guided meditation practice • Crisis scenario planning | • Complete personalized coping manual • Practice relaxation techniques daily (15 minutes) • Implement one problem-solving strategy • Identify three support resources |
| 7-8 | Relationship Strengthening Training Phase | • Rebuild and strengthen couple intimacy • Improve relationship satisfaction • Establish maintenance mechanisms | • Intimate relationship rebuilding: gradually restoring physical intimacy, rebuilding sexual confidence, enhancing emotional connection • Joint activity planning: developing pleasant activity plans for couple participation, cultivating common interests • Gratitude expression training: learning to express appreciation for partners, establishing positive interaction patterns • Future planning discussions: jointly developing health management plans, life goals, coping strategies • Relationship maintenance skills: establishing regular communication time, conflict prevention mechanisms • Outcome consolidation: reviewing intervention gains, developing self-management plans | • Couple interactive games • Intimacy-building exercises • Gratitude sharing circles • Future visioning activities • Behavioral commitment exercises • Group celebration and review | • Plan and engage in one pleasant couple activity weekly • Share daily gratitude (3 things) • Develop 3-month relationship goals • Create maintenance checklist |
